# Double disparities in the health care for people with schizophrenia of an ethnic-national minority

**DOI:** 10.1186/s13584-017-0166-z

**Published:** 2017-10-15

**Authors:** Gilad Gal, Hanan Munitz, Itzhak Levav

**Affiliations:** 1 School of Behavioral Sciences, Tel Aviv-Yaffo Academic College, Rabenu Yeruham St, Tel Aviv, Israel; 2 Clalit Health Services, Tel Aviv, Israel; 3 Department of Community Mental Health, Faculty of Social Welfare and Health Sciences, Haifa University, Haifa, Israel

## Abstract

**Background:**

Studies have shown health care disparities among persons of minority status, including in countries with universal health care. Yet, a dearth of studies have addressed disparities resulting from the combined effect of two minority status groups: severe mental illness and ethnic-national sector filiation. This study aimed to compare the differential health care of Jewish- and Arab-Israelis with schizophrenia in a country with a universal health insurance.

**Method:**

This study builds on a large case-control epidemiological sample (*N* = 50,499) of Jewish- (92.9%) and Arab-Israelis (7.1%) service users with (*n* = 16,833) and without schizophrenia (*n* = 33,666). Health services records were collected in the years 2000–2009. Diabetes and cardiovascular disease (CVD) served as sentinel diseases. We compared annual number of LDL tests and visits to specialists in the entire sample, Hemoglobin-A1C test among people diagnosed with diabetes, and cardiac surgical interventions for those diagnosed with CVD.

**Results:**

Service users with schizophrenia were less likely to meet identical indexes of care as their study counterparts: 95% of cholesterol tests (*p* < .001), and 92% visits to specialists (*p* < .001). These differences were greater among Arab- compared to Jewish-Israelis. Annual frequency of Hemoglobin-A1C test among people diagnosed with diabetes was lower (94%) in people with schizophrenia (*p* < 0.01), but no ethnic-national differences were identified. Among service users with CVD less surgical interventions were done in people with schizophrenia (70%) compared to their counterparts, with no ethnic-national disparities.

**Conclusions:**

In Israel, service users with schizophrenia fail to receive equitable levels of medical and cardiac surgical care for CVD and regular laboratory tests for diabetes. Although disparities in some health indicators were enhanced among Arab-Israelis, schizophrenia was a greater source of disparities than ethnic-national filiation.

## Background

Persons with severe mental illness (SMI), particularly among those with schizophrenia, are at increased risk for physical comorbidity and premature death [1–6]. Inequality in health care for people with SMI plays a major role in what has been termed “*the scandal of premature mortality*” [7], and served as the basis of a recent call for world action [8]. Findings on health care disparities were shown in the US, including in population groups protected by special insurance systems (e.g., Army veterans) [9–12], as well as in countries with national health insurance, where care is not dependent on out-of-pocket expenditure, e.g., Australia [4], Canada [13], Denmark [14], Israel [15], Sweden [16], and Taiwan [17]. These studies frequently focused on persons with SMI and somatic comorbidities. The rationale to study persons with chronic (e.g., cardiovascular disease (CVD) or diabetes) [12, 13, 15] or specific acute conditions (i.e., myocardial infarction) [11, 14] is that they represent sub-populations with increased health care needs, thus providing an appropriate test-case to study service provision by the health systems.

Analogously to SMI-related disparities, reports on risk factors for physical illness and inequalities in the use of health services were associated with ethnic-national filiation. In Israel, Arab-Israelis were found to be at higher risk for obesity [18, 19], diabetes [20, 21], CVD [22], and show earlier age of heart failure and diabetes presentation [18]. In addition, although Israel has a national health insurance where care is not dependent on out-of-pocket expenditure, inequalities in health care between Jewish- and Arab-Israelis were reported. Visits to general practitioners (GPs) were found to be higher, while visits to specialists lower, among Arab- compared to Jewish-Israelis [23]. Among people diagnosed with diabetes less effective disease control was reported in Arab- compared to Jewish-Israelis [21]. Thus, similarly to persons with SMI, the combination of increased morbidity risk and lower quality of health care was shown among Arab-Israelis.

The above brief review raises an intriguing question, is there a compounded health risk effect among Arab-Israelis diagnosed with SMI? A recent epidemiological study in the UK reported an additive risk for diabetes among ethnic minorities in the presence of SMI [24]. Thus, it is possible that two additive effects also result in less health care. Today the confirmatory answer to this question is pending. We have found only one population-based study, which showed that Hispanics diagnosed with schizophrenia in the US have worse treatment rates for metabolic abnormalities (i.e., diabetes, hypertension) when compared to non-Hispanics [25]. However, this study relied on a database selective for people with schizophrenia and did not compare the findings with the general population.

In light of the above, we hypothesized that individuals with schizophrenia would have lower rates of medical care than those without schizophrenia. In addition, we expected to find greater disparities among individuals with schizophrenia in Arab- compared to Jewish-Israelis.

### Objective

To compare health care disparities between Jewish- and Arab-Israelis with schizophrenia in a country with national health insurance. Comorbid CVD and/or diabetes were chosen as sentinel diagnoses.

## Methods

This study was based on a prospective-historical design with data collected between the years 2000–2009. Ethical approval was granted by the head management of the health provider, *Clalit Health Services* (CHS). Participants’ identity was undisclosed to the authors.

### Identification of persons with schizophrenia

Psychiatric care in Israel is freely available by law to all *de-jure* residents [26]. To identify the sample of persons with schizophrenia we used the National Psychiatric Case Register (NPCR). The NPCR is legally mandated to maintain a cumulative record of psychiatric hospitalizations in mental and general hospitals [27, 28] and includes data on all the individuals who have had at least one psychiatric hospitalization. The NPCR provided the subjects’ socio-demographic information and psychiatric diagnoses upon admission and discharge. The latter diagnosis recorded during the last admission was used in this study, following the assumption that the longer the period of observation the higher the reliability. Diagnoses were based on ICD-10 [29], those made prior to its introduction have been updated. Tests of agreement between research and NPCR diagnoses were found satisfactory [30, 31]. The following inclusion criteria were applied: a. persons with last discharge diagnosis of schizophrenia (F20-F29); b. born prior to 1960 (age > 40 at the start of the follow up period); and c. alive by the year 2000. The reason for the age criterion was to include older service users that generally are in greater need for non-communicable diseases-related health services.

We identified 28,579 persons who met the inclusion criteria in the NPCR records. The information gathered included: year of first and last psychiatric hospitalization, total number of hospitalizations, and total length of hospital stay (Table 1). The latter was recorded annually (years 2000–2009) to control for possible artifacts, since, while in hospitalization the service user would not be under the care of CHS, unless referred by the treating doctors in the mental hospitals.

**Table 1:** Socio-demographic characteristics and clinical data of service users diagnosed with schizophrenia and matched controls

	Schizophrenia (*n* = 16,833)	Matched controls (*n* = 33,666)
Gender (M)	49.3%	49.3%
Age (2009)^a^	63.0 (9.2)	63.0 (9.2)
Birthplace		
Israel	42.0%	42.0%
Asia	14.7%	14.7%
Africa	17.3%	17.3%
Europe-America	26.0%	26.0%
National-sector		
Jewish-Israelis	92.9%	92.9%
Socioeconomic status		
Low	39.9%	39.9%
Middle	44.0%	44.0%
High	16.1%	16.1%
Age at first hospitalization^a^	33.7 (13.7)	--
Number of psychiatric hospitalizations^a, b^	7.2 (9.2); 4.0	--
Annual hospitalization days (2000–2009)^a^	15.8 (41.2)	--

^a^Mean (SD)

^b^Median

### General health services

Data from CHS, Israel’s largest health maintenance organization (HMO), with 3.8 million insured persons, were extracted from its electronic database. The sampling frame was defined according to the above-noted second inclusion criterion (i.e., age), and comprised of 1,040,000 individuals. Data on the cohort were gathered annually during the years 2000–2009, however some of the data were available from the year 2002 onwards when the CHS initiated a central data base.

The following data were collected:

1. Socio-demographic data: Sex, year and place of birth, ethnic-national sector (Jewish- and Arab-Israelis), year of death, and socioeconomic status. Since personal data on ethnicity are not kept in the health maintenance organization information system, Arab-Israelis were identified by their place of residence or in large mixed cities (e.g., Haifa, Nazareth) by the sub-district. Socioeconomic status was based on information gathered from the National Central Bureau of Statistics on average income levels according to geographical data. We used the geographical location of the clinic as a proxy for the income and hence the socioeconomic rank of the user: low (income deciles 1–3), middle (deciles 4–7), and high (deciles 8–10).
2. Medical diagnoses: The cardiovascular ICD-10 diagnoses included were: ischemic heart disease (IHD) (I20-I25); congestive heart failure (CHF)(I50); cardiomyopathy (I42); idiopathic hypertrophic subaortic stenosis (IHSS)(I42.1-I42.5); pulmonary hypertension (I27); and carotid artery disease (I65.2). In addition, type 2 diabetes mellitus (E11) was recorded. These were extracted from the service users’ files, as well as the respective dates of the diagnoses.
3. Laboratory tests: The number of the following tests were recorded annually: blood cholesterol fraction tests (LDL) (2002–2009); Hemoglobin-A1C tests (2002–2009); and visits to specialists (2000–2009). In addition, the minimal and maximal LDL cholesterol and Hemoglobin-A1C levels were recorded annually (2002–2009). For each measure the highest annual level was used. Hemoglobin-A1C is measured primarily to identify the average plasma glucose concentration over prolonged periods and is required in the use of second-generation anti-psychotic medications because of the associated risk for diabetes. There are several reasons to assume that the service users in our study would do Hemoglobin-A1C and LDL tests. First, since the study participants were relatively aged it is reasonable to assume that they do undergo physical tests. Second, those tests are part of the National Quality Indicators Program (NQIP), promoted by the Israeli Ministry of Health, as a result of the increase awareness of preventive medicine in the context of the metabolic syndrome and the diagnosis of diabetes. However, while annual Hemoglobin-A1C test is recommended for persons diagnosed with diabetes, no clear recommendation has been made for people of the general population that are diagnosis free.
4. Visits to specialists: number of annual visits, excluding psychiatrists (2000–2009).
5. Cardiac surgical interventions: For service users diagnosed with CVD catheterization, coronary artery bypass graft (CABG) and pacemaker implantation performed between the years 2000–2009 were extracted from the service users’ files, as well as the dates the interventions were conducted.

### Linkage procedure and selection of a matched control group

The data extracted from the NPCR and CHS databases were merged according to the personal ID. To generate a matched control group (2:1 ratio) an algorithm was defined based on age (groups of three consecutive years), sex, birth continent (Israel, Asia, Africa, Europe-America), socioeconomic status (high, middle, low) and ethnic-national sector (Arab- and Jewish-Israelis). If the service user with schizophrenia had missing information on one of the matching variables, controls with missing data on that same variable were chosen. Service users were omitted in case of: death that occurred before 2002, and an average annual psychiatric hospitalization of 270 days or more. The matching procedure yielded 16,833 service users diagnosed with schizophrenia, and 33,666 matched controls, and was found satisfactory (Table 1). The ethnic-national sectors comprised of 15,641 Jewish- (92.9%) and 1192 Arab-Israelis (7.1%) diagnosed with schizophrenia, and their matched controls (31,282; and 2384, respectively).

Further inclusion criteria were applied to persons diagnosed with CVD: a. the first CVD diagnosis had been made during the follow-up period (2000–2009); and b. the first psychiatric hospitalization of service users with schizophrenia preceded the first CVD diagnosis.

### Data analysis

The associations between psychiatric diagnosis (schizophrenia vs. comparison) and ethnic-national sector (Jewish- vs. Arab-Israelis) with laboratory tests and visits to specialists were analyzed using general linear models (GLM): analysis of variance (ANOVA), and logistic regression. Rates of diabetes, CVD and cardiac surgical interventions were analyzed using logistic regression models. Statistical outcomes of the logistic regression tests were estimated using odds ratios (OR) and 95% confidence intervals (95% CI). In addition, surgical interventions and mortality were analyzed using Cox proportional-hazards models. Statistical outcomes of Cox-regression tests were estimated using hazard ratios (HR) and 95% CI. Cardiac surgical interventions were calculated accounting for the relevant years of follow-up since the CVD diagnosis (min = 1, max = 10). Univariate analysis was conducted for all models to test the association between medical procedures conducted and potential confounders, e.g., sex, age, and socioeconomic status. The adjusted analysis of surgical interventions accounted, in addition, for the age at first CVD diagnosis. Adjusted analysis followed the univariate analysis to include confounding variables showing significant univariate associations with the outcome variables. Analysis was performed using the SPSS 21.0 software (IBM Inc.)

## Results

Less annual LDL tests were recorded for service users diagnosed with schizophrenia than among their counterparts, particularly for Arab- compared to Jewish-Israelis (Table 2). The analysis of annual LDL tests indicated a significant main effect of diagnosis (adjusted F = 36.2, df = 1, 46,714, *p* < 0.001), and a significant diagnosis by ethnic-national sector interaction (adjusted F = 6.6, df = 1, 46,714, *p* < 0.01). The mean LDL level was slightly lower among Arab-Israelis with schizophrenia (120.9 ± 28.9) (mean ± SD) than their comparison group (123.9 ± 28.4), but not among Jewish-Israelis (schizophrenia 126.9 ± 27.8; comparison 126.4 ± 29.7). This was supported by a significant diagnosis by ethnic-national sector interaction (adjusted F = 4.5, df = 1, 43,609, *p* = 0.03).

**Table 2:** Annual rate (mean, SD) of LDL tests and visits to specialists among Jewish- and Arab-Israelis with schizophrenia and matched controls

	Jewish Israelis		Arab Israelis	
	Schizophrenia	Matched controls	Schizophrenia	Matched controls
LDL test	0.95 (0.8)	1.00 (0.8)	0.77 (0.6)	0.88 (0.7)
Visits	2.51 (3.4)	2.65 (2.9)	1.50 (2.2)	1.83 (2.1)

People diagnosed with diabetes did hemoglobin-A1C test predominantly: throughout the follow-up period, 84.6% of the service users diagnosed with diabetes had at least one Hemoglobin-A1C test compared to 8.9% of those who were not diagnosed. This implies the existence of two sub-populations with regard to Hemoglobin-A1C test, namely, service users diagnosed or not diagnosed with diabetes. Therefore, we analyzed the annual Hemoglobin-A1C test only among people diagnosed with diabetes (*n* = 12,657; 31.6% of service users with schizophrenia and 27.0% of comparisons). Throughout the follow-up period, 84.6% of the service users with schizophrenia and 83.7% of comparisons did at least one Hemoglobin-A1C tests. However, less annual Hemoglobin-A1C tests were done by service users with schizophrenia (1.83 ± 1.9) than counterparts (1.95 ± 1.8) (adjusted F = 13.9, df = 1, 10,405, *p* < 0.001). No difference was found between ethnic-national sectors.

Less annual visits to specialists were recorded for service users diagnosed with schizophrenia than among their counterparts, and lower among Arab- compared to Jewish-Israelis (Table 2). In addition, greater difference of visits to specialists were found between service users diagnosed with schizophrenia and their counterparts in the Arab- compared to the Jewish-Israeli sector. The analysis of visits to specialists indicated the significant main effects of diagnosis (adjusted F = 23.3, df = 1, 49,588, *p* < 0.001) and ethnic-national sector (adjusted F = 133.5, df = 1, 49,588, *p* < 0.001), as well as a significant diagnosis by ethnic-national sector interaction (adjusted F = 5.4, df = 1, 49,588, *p* = 0.02).

During the follow-up period, death records were noted in 7192 (14.2%) service users. Mortality rate was 2.3 fold higher among service users with schizophrenia (22.5%) compared to matched controls (10.1%) (adjusted HR 2.30, 95% CI 2.13–2.49). Mortality rates did not differ between Jewish- and Arab-Israelis.

### Service users diagnosed with CVD

CVD diagnosis was found in 7920 service users: schizophrenia, 2224 (13.2%); matched controls, 5696 (16.9%). Among service users with schizophrenia the rate of CVD diagnosis did not differ between Jewish- and Arab-Israelis (13.1 and 14.1%, respectively). Age at first CVD diagnosis was slightly lower among service users with schizophrenia (60.8 ± 9.1) compared to controls (62.7 ± 9.1)(F = 4.4, df = 1,7693, *p* = 0.037), and among Arab- (58.9 ± 8.5) compared to Jews-Israelis (62.9 ± 9.1)(F = 4.4, df = 1,7693, *p* = 0.037).

We identified 3041 service users with CVD who underwent cardiac surgical interventions (38.4%). Lower rates of catheterization, CABG and pacemaker implantation were recorded among service users with schizophrenia compared to matched controls (Table 3). Although rates tended to be higher among Arab- compared to Jewish-Israelis, no significant associations were found. The total rate of cardiac surgical interventions among service users with schizophrenia was 29.1% compared to 42.0% in their counterparts. Adjusted Cox-regression model indicated that service users with schizophrenia had 30% decreased likelihood of surgical interventions than matched controls (adjusted HR 0.70, 95% CI 0.64–0.76).

**Table 3:** Rates of cardiac surgical interventions among Jewish- and Arab-Israelis with schizophrenia and matched controls diagnosed with a cardiovascular disease

	Jewish Israelis		Arab Israelis		
	Schizophrenia	Matched controls	Schizophrenia	Matched controls	OR (95% CI)^a^
Cardiac catheterization	21.6%	32.3%	26.6%	37.8%	**SMI** 0.57 (0.51–0.64) **Sector** 0.95 (0.80–1.14)
Coronary artery bypass graft	8.2%	11.1%	10.7%	15.4%	**SMI** 0.72 (0.61–0.86) **Sector** 0.81 (0.63–1.03)
Cardiac pacemaker implantation	1.4%	2.3%	1.2%	2.0%	**SMI** 0.60 (0.40–0.90) **Sector** 0.82 (0.44–1.53)
Any intervention	28.7%	41.3%	34.9%	49.1%	**SMI** 0.56 (0.50–0.62) **Sector** 0.87 (0.73–1.04)

^a^Adjusted model included sex, age, socioeconomic status, and age at first cardiovascular diagnosis

During the follow-up period death records were noted in 2123 service users with CVD diagnosis. Following the CVD diagnosis the mortality rate was 2.3 fold higher in service users with schizophrenia compared to their counterparts (adjusted HR 2.34, 95% CI 2.14–2.56). Death risk was reduced by more than 50% among service users who underwent a cardiac surgical intervention (adjusted HR 2.28, 95% CI 2.05–2.54). Death rates did not differ between Jewish- and Arab-Israelis.

## Discussion

In keeping with best practice in the study of health care disparities, our study sought “To gain insight into the performance of health care providers and systems” and thus to obtain high returns for service users and providers [32]. Results identified health services disparities among persons with schizophrenia. Lower rates of laboratory tests and visits to specialists were found as well as additive effects for Arab-Israelis with diagnosed schizophrenia. Less cardiac surgical interventions were found among service users with comorbid schizophrenia and CVD compared to matched controls. Elevated death risk was seen among persons with schizophrenia of both ethnic-national sectors.

Based on previous findings of health care disparities among service users with schizophrenia [15] together with reports on inequalities to Arab-Israelis [21, 23] we hypothesized additive effects of disparities among Arab-Israelis with schizophrenia. This hypothesis was mostly unconfirmed. Additive effects were found with regard to two measures, rate of annual LDL tests and visits to specialists. With regard to both measures less health care was found among service users with schizophrenia than matched controls and the difference was enhanced among Arab-Israelis.

A pattern of reduced use of specialized health services together with an increased use of GPs’ services among Arab-Israelis has been reported previously [23] as well as among other groups of minorities in the US [33]. Similar results were noted for people of lower socioeconomic status in high income countries [34, 35]. This pattern of service use was explained by accessibility issues [23]. While most specialist physicians are located in metropolitan areas, the majority of Arab-Israelis live in small towns and villages, and it may be more difficult for them to access specialist care compared with the Jewish-Israelis that reside mostly in larger cities. As the GPs are more available in the near vicinity of their home they tend to substitute visits to specialists by greater use of the former. Due to the functional difficulties of people with schizophrenia, accessibility factors play a greater role as a barrier of health services use thus intensifying this reduced pattern of visits to specialist physicians.

The findings of the current study indicate that Arab- did significantly less LDL tests compared to Jewish-Israelis. As performance of LDL test is related to risk for diabetes and CVD which is higher among Arab- compared to Jewish-Israelis [18, 20], this finding calls for the health maintenance organizations attention. A tendency to less adequate LDL control among Arab-Israelis diagnosed with diabetes was reported already [21], but findings with regard to the general population (i.e., including undiagnosed people) are not available.

Service users with diabetes in our study received equitable health care regardless of the presence of schizophrenia and ethnic-national filiation. People with schizophrenia had lower frequency of annual Hemoglobin-A1C tests, but similar rate of people did A1C test throughout the follow-up period. Furthermore, people with schizophrenia had lower Hemoglobin-A1C level than their counterparts. Similar to previous findings no differences between Jewish- and Arab-Israelis in the frequency of annual Hemoglobin-A1C tests were noted [21].

The increased rate of diabetes amongst service users with schizophrenia adds to the burden carried by this vulnerable population [36, 37]. It is possible that the equality of care we found may be attributed to the availability, accessibility and the quality of the specialized services in Israel. Specifically, according to the NQIP, the treatment of persons with diabetes should follow current standards of medical care [38] including annual Hemoglobin-A1C and LDL tests and the use of anti-diabetic medications. The CHS has implemented the NQIP’s recommendations since 2001 in both primary care and specialized diabetes clinics, enabling improved follow-up and case management. The positive outcomes of the program is supported by our study. Importantly, these services are facilitated by the National Health Insurance that grants all service users free and direct care. Plausibly, these reduced disparities benefited from the specific services mandated by the rehabilitation law adopted over a decade ago for service users with mental disabilities [39–41].

In contrast, disparity in cardiac surgical interventions among service users with CVD was observed. Performance of interventions was lower in service users with schizophrenia compared to their counterparts. The chance of service users with schizophrenia to receive any type of cardiac surgical intervention was found to be 30% than counterparts, indicating a major disparity affecting the former population. However, contrary to our hypothesis a tendency to more frequent performance of catheterization and CABG among was recorded in Arab- compared to Jewish- Israelis. Notably, CVD was diagnosed four years earlier in Arab- compared to Jewish-Israelis, an effect that could be attributed to an earlier age of onset of CVD among the former (18). Conceivably, as the tendency for surgical intervention following CVD diagnosis is higher among younger patients [42], the age factor could explain the slightly higher frequency of surgical interventions in this group.

Accumulative evidence has pointed out that risk factors for CVD are more common in persons with schizophrenia [43, 44]. Along with excess in mortality, this may suggest that persons with schizophrenia should have higher rates of somatic care and cardiac surgical interventions, yet we found lower rates of surgical interventions following CVD diagnosis. A possible explanation may argue that symptoms of heart disease were fewer among persons with schizophrenia. However, the same pattern emerged when we examined specific sub-groups of patients with multiple CVD diagnoses or with comorbid diabetes in which more severe symptoms could be assumed. A similar observation was reported in people with SMI following myocardial infarction [14].

This study has several limitations. First, the data are based on the performance of the medical procedures rather than on the examination of doctors’ prescriptions. Therefore, we could not differentiate between the actions of the treating physicians, possibly based on stigma [45] and the users’ adherence to care. This distinction is important with regard to service users with schizophrenia since compared to the general population their clinical condition may compromise their health behavior. Second, we did not have access to information on risk factors (e.g., smoking and obesity). Third, the rate of CVD was slightly lower among persons with schizophrenia than in the comparison population. This apparently surprising finding could be explained by a synergy between competing risks and age factors in our sample. Thus, premature mortality (all subjects were 40+ at the start of the follow up period) could remove persons with schizophrenia from the at-risk population, leading to reduced CVD prevalence. A reduced risk in morbidity as age increases has been reported in the literature [46, 47]. Last, we had no information on the severity of both the psychiatric and the physical illness involved. This is a common limitation of epidemiological studies that rely on administrative data-bases. We think, however, that the limitations are balanced in part by the large size of the populations investigated, the fact that the same health care provider (CHS) served the entire sample, the varied types of the measures used, the careful recording of the information collected on the users, and the consistency of the results.

### Policy implications

A population-based study [1] that explored the mortality risk for persons with SMI in Israel found that the adjusted rates for 100,000 persons for Israeli Jews aged 18 and above compared with non-hospitalized subjects in the years 1981–2006 were 1.6 for CVD and 2.1 for diabetes. An additional epidemiologic study based on Israel’s second largest HMO reported on an standardized mortality rate of 2.4 among persons diagnosed with schizophrenia [48]. Although the findings of these studies clearly established a case for action, no comprehensive programs of health promotion and disease prevention have been implemented in Israel to reduce such a risk. Acutely cognizant of the problem world-wide, both in terms of risk and efforts to reduce it, as well as of countries that implemented programs of action (e.g., Australia), the World Health Organization has recommended “a multilevel framework of intervention” that comprises of three components: individual, health-system and socio-environmental [49]. The segment covered in the current study was the health-system component. The purpose, as noted in the introduction, was to explore whether it operated flawlessly, accounting for possible barriers raised by stigma and cognitive deficits associated with schizophrenia [49].

The disparities found constitute a limited observance of the United Nations Convention on the Rights of Persons with Disabilities (CRPD) (United Nations, 2006). Importantly, CRPD establishes to *“Provide persons with disabilities with the same range, quality and standard of free or affordable health care and programs as provided to other persons…” (Art. 25)*. Clearly, and despite the binding nature of CRPD for the signatory countries, in Israel, as in other countries with universal health insurance, persons with schizophrenia do not benefit from equal specialized medical care compared to persons free of this disorder. This disparity requires special attention by providers since the use of atypical antipsychotic drugs may cause overweight and diabetes, both of which are risk factors for CVD [44, 50].

Insel & Landis noted that *“The public health challenge* [in mental health care] *is mortality as well as morbidity”* [51]. Innovative and complementary strategies, as proposed by WHO (see above), are needed to correct deficiencies in the medical practice as well as in the engagement of persons with schizophrenia, their families and the service users associations, in the development of programs of health promotion, primary prevention and curative care [52–54].

## Conclusion

In Israel, despite the existence of universal health insurance, service users with schizophrenia fail to receive equitable levels of medical care. Additive disparity among Arab-Israelis with schizophrenia was related to specific health-care indicators only. Schizophrenia was found to be a more marked source of disparities than ethnic-national filiation.

## Abbreviations

CABG: Coronary artery bypass graft
CHS: Clalit Health Services
CRPD: Convention on the Rights of Persons with Disabilities
CVD: Cardiovascular disease
GP: General practitioners
HMO: Health Maintenance Organization
NPCR: National Psychiatric Case Register
NQIP: National Quality Indicators Program
SMI: Severe mental illness
